# The Changes of Microbial Communities and Key Metabolites after Early *Bursaphelenchus xylophilus* Invasion of *Pinus massoniana*

**DOI:** 10.3390/plants11212849

**Published:** 2022-10-26

**Authors:** Yibo An, Yongxia Li, Ling Ma, Dongzhen Li, Wei Zhang, Yuqian Feng, Zhenkai Liu, Xuan Wang, Xiaojian Wen, Xingyao Zhang

**Affiliations:** 1 College of Forestry, Northeast Forestry University, Harbin 150040, China; 2 Key Laboratory of Forest Protection, National Forestry and Grassland Administration, Ecology and Nature Conservation Institute, Chinese Academy of Forestry, Beijing 100091, China; 3 Co-Innovation Center for Sustainable Forestry in Southern China, Nanjing Forestry University, Nanjing 210037, China

**Keywords:** pinewood nematode, microbial community, endophytic microbes, metabolite profiling, pines

## Abstract

Pine wood nematode, *Bursaphelenchus xylophilus*, is a worldwide pest of pine trees, spreading at an alarming rate and with great ecological adaptability. In the process of causing disease, the nematode causes metabolic disorders and changes in the endophytic microbial community of the pine tree. However, the changes at the pine nidus during early nematode invasion have not been well studied, especially the differential metabolites, in *Pinus massoniana*, the main host of *B. xylophilus* in China. In this study, we analyzed the endophytic bacterial and fungal communities associated with healthy and *B. xylophilus*-caused wilted pine trees. The results show that 1333 bacterial OTUs and 502 fungal OTUs were annotated from *P. massoniana* stem samples. The abundance of bacterial communities in pine trees varies more following infection by *B. xylophilus*, but the abundance changes of fungal communities are less visible. There were significant differences in endophytic microbial diversity between wilted and healthy *P. massoniana*. In wilted pine trees, Actinobacteria and Bacteroidia were differential indicators of bacterial communities, whereas, in healthy pine trees, *Rhizobiales* in the *Proteobacteria* phylum were the major markers of bacterial communities. Meanwhile, the differential markers of fungal communities in healthy pines are *Malasseziales*, *Tremellales*, *Sordariales*, and *Fusarium*, whereas *Pleosporaceae* is the key marker of fungal communities in wilted pines. Our study examines the effect of changes in the endophytic microbial community on the health of pine trees that may be caused by *B. xylophilus* infection. In parallel, a non-targeted metabolomic study based on liquid mass spectrometry (LC-MS) technology was conducted on pine trees inoculated with pine nematodes and healthy pine trees with a view to identifying key compounds affecting early pine lesions. Ultimately, 307 distinctly different metabolites were identified. Among them, the riboflavin metabolic pathway in pine trees may play a key role in the early pathogenesis of pine wood nematode disease.

## 1. Introduction

Pine wilt disease (PWD) causes massive damage to ecosystems and is now a worldwide threat to forest ecology and international trade [1,2], the pathogen of which is pine wood nematode (*Bursaphelenchus xylophilus*), a migratory endoparasitic nematode. Regarding the pathogenic mechanism of pine wood nematodes, there are currently three main theories, namely: 1. Enzyme theory: some scholars have extracted cellulose from the worm body pulp and exudate of pine wood nematodes, which causes the cell wall of pine parenchyma cells to become enzymatically hydrolyzed [3]. It is suggested that the cause of pine wilting may be due to various enzymes secreted by *B. xylophilus* that are harmful to the parenchyma cells so the parenchyma cells cannot function normally [4]. As a result, the absorption of water and nutrients by the pine tree is hindered, and eventually the pine tree wilts; 2. Hollow chemistry theory: The chemical defense mechanism of plants can secrete and synthesize some metabolites to help themselves avoid stress when they receive external stress damage. Researchers have found that pinene-like compounds are abundantly secreted in pine trees following *B. xylophilus* infestation and colonization [5,6]. These substances are highly hydrophobic and easily vaporize. At this time, for such substances, the pine tree cannot degrade itself and will penetrate into the tracheids of the xylem to form cavities, resulting in the inability of the pine tree to transport water, and eventually, the pine tree will die due to lack of water; 3. Toxin theory: Some researchers believe that toxins may come from abnormal metabolites of the pine host, such as benzoic acid, 8-hydroxycarvone, catechol, etc. [7,8,9]. Another section of the researchers believes that it may be the toxins produced by the accompanying bacteria of *B. xylophilus*, such as: chelating ferritin, wilting toxin, and so on [10,11].

Specific bacteria associated with *B. xylophilus* have been reported to play an important role in the pathogenesis of PWD [12,13]. Among them, specific bacteria will produce corresponding toxins to poison the host pine trees, such as phenylacetic acid [7,12]. The bacteria carried by nematodes exist as long-term co-evolved symbionts and are not accidentally contaminated; for example, the highly pathogenic *Pseudomonas fluorescens* GcM5-1A and *Pseudomonas putida* ZpB1-2A can significantly increase the fecundity, reproduction rate, and size of *B. xylophilus* [14]. In contrast, some microbes protect pines from PWDs such as *Esteya* [15], *Serratia* [16], and *Trichoderma* [17]. Although *Pinus massoniana* is widely distributed in central and southern China [18,19], the microbial community after *B. xylophilus* infection has not been well studied, especially its fungal community. Previous studies have shown that the enrichment of differential genes was significant when *B. xylophilus* infected the host for 15 days [20]. Some researchers found that the chemical defense substance α-pinene in *P. massoniana* was abundantly enriched when *B. xylophilus* infested the *P. massoniana* for 15 days [21]. Therefore, no one has studied the fixed time node of host onset in the pathogenesis of *B. xylophilus*. This work used high-throughput Illumina MiSeq (Illumina, San Diego, CA, USA) to discover changes in host–microbial communities that may be caused by PWDs at specific time points and to clarify the relationship between *B. xylophilus* and host–microbial communities.

Plants are capable of producing a variety of secondary metabolites that act as toxins and prevent feeding by mammals and insects [22]. For example, monoterpenes in Gymnosperms, such as Pine, accumulate in resinous vessels found in branches, trunks, and needles, mainly α-pinene, β-pinene, myrcene, and limonene. These plant metabolites are toxic to a variety of insects, including bark beetles, a serious pest of gymnosperms worldwide [5]. *B. xylophilus* also affected changes in *P. massoniana* metabolites. Changes in metabolites also affect pathogenic processes. The relationship between the microorganisms, nematodes, and multi-species metabolites of pine wood nematodes is thus complicated, and the relationship between the metabolites and microorganisms in the pathogenic process is still unclear.

Based on the cavitation hypothesis [5], the abnormal accumulation of metabolites in pine trees is likely to be the main reason for the cavitation of tracheids. Therefore, the sampling phase of this study selected a critical time point in previous studies, that is, 15 days after the pine wood nematode-infested pine trees. At the same time, for the sampling site, we selected the stem in the semi-withered state as the research object, that is, the junction of withered and non-withered tissues, as the test material of this study.

The purpose is to pay attention to the changes in the microecological environment of pine trees at key nodes in the pathogenic process, such as the abnormal accumulation of metabolites and obvious disease phenotypes. The effects of *B. xylophilus* on the host were discussed from the perspectives of microbial community changes and host metabolism. By correlating microbial community alterations with metabolite variations, it is intended to bring new ideas and supplements for the pathogenic mechanism of *B. xylophilus*.

## 2. Result

### 2.1. Microbial Richness and Diversity Indices

**Figure 1:**
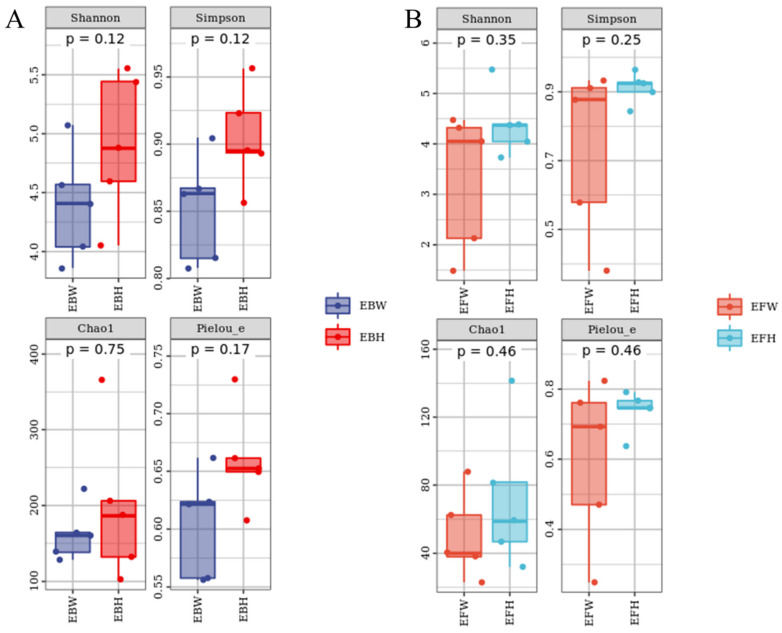
Alpha Diversity Index of the microbial communities of healthy and *B. xylophilus*–infected *P. massoniana*. (**A**) Endophytic bacteria. (**B**) Endophytic fungi. Nematode-infected Wilted pines are designated as *P. massoniana*. EBH, healthy pine endophytic bacteria; EBW, wilted pine endophytic bacteria; EFH, healthy pine endophytic fungi; EFW, wilted pine endophytic fungi.

From *P. massoniana* stem tissues, we collected 111,951 bacteria reads and 496,360 fungal reads, respectively. A total of 1333 bacterial OTUs and 502 fungal OTUs were assigned to these sequences, respectively. The Chao1 [23] index (Figure 1) and the reads obtained from the SPSS software analysis of each sample (Table 1) jointly characterized the richness of the microbial community in the pine trees and found no significant difference in the richness of the endophytic microbial communities between wilted (*B. xylophilus*-infected) pines and healthy pines. Although not significant, the richness and diversity of the microbial community in the healthy pines were higher than those in the wilted pines. At the same time, the Shannon [24] and Simpson [25] indices were used to determine the diversity, and the Pielou’s [26] evenness index was used to determine the uniformity, and no significant variations in the diversity and uniformity of endophytic microbial communities were discovered between the wilted pine trees and the healthy pine trees (Figure 1).

### 2.2. Diversity of Microbial Composition

**Figure 2:**
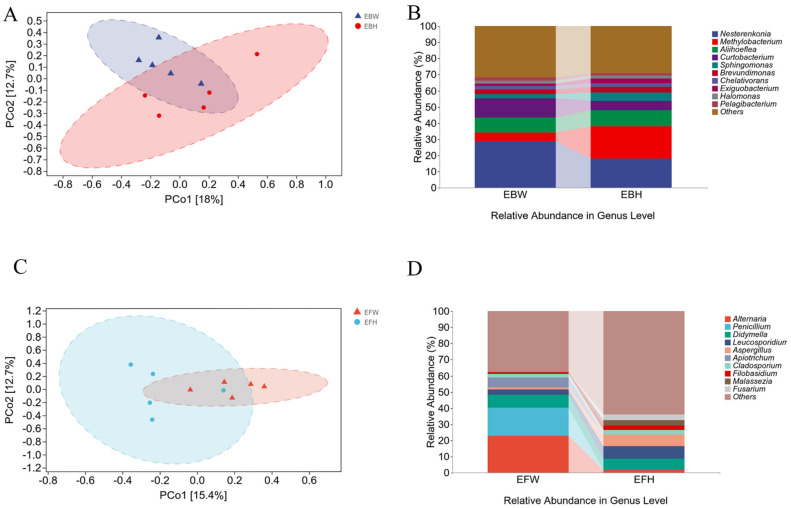
Clustering of the microbial communities of healthy and *B. xylophilus*−infected *P. massoniana* using PCoA and UPGMA (unweighted pair-group method with arithmetic means). (**A**) PCoA plots based on Bray–Curtis metrics for bacterial communities of healthy and *B. xylophilus*−infected *P. massoniana*. (**B**) UPGMA clustering of bacterial communities of healthy and *B. xylophilus*−infected *P. massoniana*. (**C**) PCoA plots based on unweighted Bray–Curtis metrics for fungal communities of healthy and *B. xylophilus*−infected *P. massoniana*. (**D**) UPGMA clustering of fungal communities of healthy and *B. xylophilus*−infected *P. massoniana*.

pCoA (Figure 2A,C) and UPGMA (Figure 2B,D) sequencing analyses were performed based on OTU detected in the samples (showing the 10 species with the highest relative abundance; the remaining species with relative abundance were pooled and classified as Others). The findings revealed that there was no significant difference in the endophytic microbial populations between the healthy and wilted pines (Figure 2A,C). The findings revealed that the abundance of endophytic bacterial communities in the wilted pine trees and the healthy pine trees was similar (Figure 2B). In the endophytic fungal communities, the abundance of the wilted pines was substantially higher than that of the healthy pine trees (Figure 2D). The foregoing findings imply that pine wood nematode infection has a major impact on endophytic fungal communities in pine trees, but not on bacterial communities.

### 2.3. Unique and Shared OTUs and the Key Marker OTU

**Figure 3:**
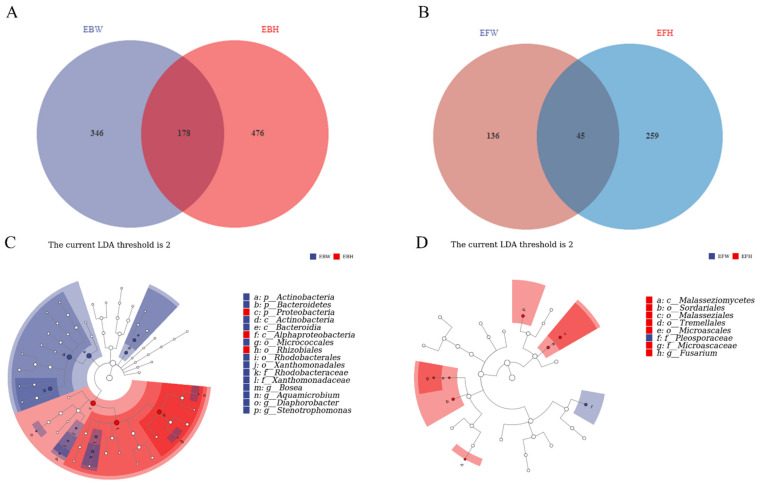
Venn diagrams of the unique and shared operational taxonomic units (OTUs) of sequenced samples and LEfSe diagrams between all the samples from healthy pines and wilted pines. (**A**) Bacterial data among all samples from healthy pines and wilted pines. (**B**) Fungal data from all the samples from healthy pines and wilted pines. (**C**) LEfSe analysis diagrams of bacteria data between all the samples from healthy pines and wilted pines. (**D**) LEfSe analysis diagrams of fungal data between all the samples from healthy pines and wilted pines.

The endophytic bacteria of healthy pines (EBH), the endophytic bacteria of wilted pines (EBW), the endophytic fungal of healthy pines (EFH), and the endophytic fungal of wilted pines (EFW) were used to split the total samples (EFW). There were 178 common bacterial OTUs between the wilted (EBW) and the healthy (EBH) pines among the *B. xylophilus*-infected samples (Figure 3A). However, both groups possessed OTUs that were unique to them, with 476 in the healthy pines and 346 in the wilted pines, resulting in a reduction in bacterial diversity in the *B. xylophilus*-infected pines. Meanwhile, there were 45 common fungal OTUs between the wilted (EFW) and healthy (EFH) pines among *B. xylophilus*-infected samples (Figure 3C). Yet, both groups possessed OTUs that were unique to them, with 259 in the healthy pines and 136 in the wilted pines, resulting in a reduction in the fungal diversity of the *B. xylophilus*-infected pines.

We used LEfSe (LDA Effect Size) analysis on both groups to determine the primary characteristics that separate healthy and wilted pine communities. The findings revealed that in wilted pine trees, Actinobacteria and Bacteroidia were differential indicators of bacterial communities, whereas, in healthy pine trees, *Rhizobiales* in the *Proteobacteria* phylum were the major markers of bacterial communities (Figure 3B). Meanwhile, the differential markers of fungal communities in healthy pines are *Malasseziales*, *Tremellales*, *Sordariales*, and *Fusarium*, whereas *Pleosporaceae* is the key marker of fungal communities in wilted pines (Figure 3D).

### 2.4. Difference Analysis between Metabolite Groups

**Figure 4:**
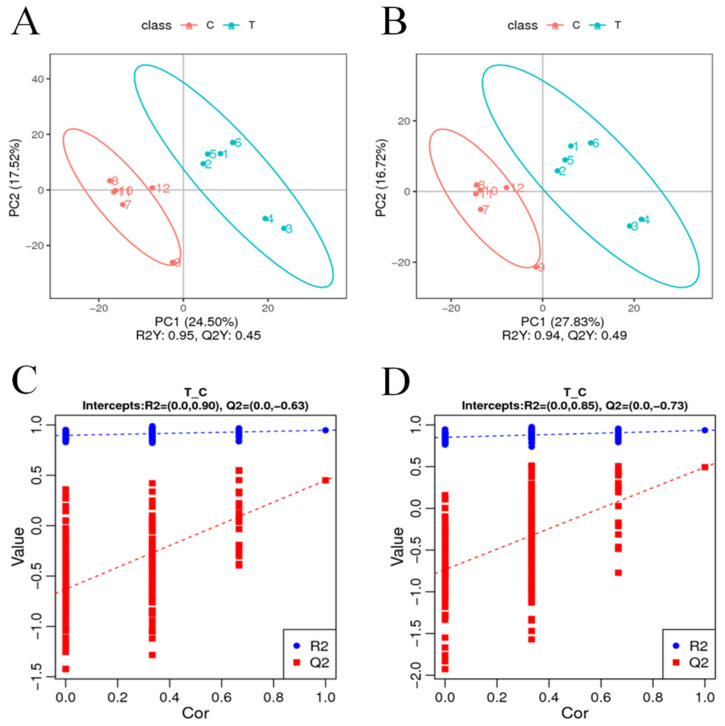
PCA analysis of differential metabolites. The horizontal coordinate PC1 and the vertical coordinate PC2 indicate the scores of the first and second principal components, while different colors indicate samples from different treatments, and the confidence ellipse is 95%. (**C**,**D**) PLS-DA classification validation plots. The horizontal coordinates indicate the correlation between random group Y and original group Y, while the vertical coordinates indicate the scores of R2 and Q2. (**A**,**C**) positive model, (**B**,**D**) negative model.

To visualize the variability and overall distribution trends between and within sample groups, PCA analysis was performed on 980 differential metabolites detected by cationic patterns in the treatment group (T) and the control group (C). The first and second principal components explained 24.50% and 17.52% of the total variation, respectively. As can be seen from the PCA plots, the T and C samples were concentrated in their respective groups, and there was a relatively clear separation between the two groups (Figure 4A). PCA analysis of the 690 differential metabolites detected in the anion pattern of each sample also showed the same trending distribution results (Figure 4B), with PCA analysis revealing objective differences in the metabolites between the T and C groups. The PLS-DA model was then constructed for the comparison group in this study, and the model evaluation parameters (R2, Q2) were obtained by 7-fold cross-validation (seven cycles of cross-validation, k cycles of cross-validation when the number of biological replicates of the sample is n <= 3, k = 2n); if R2 and Q2 are closer to 1, it indicates that the model is more stable and reliable, subsequently seen in this study. The model was then validated for overfitting, and it was found that neither model in this study was overfitted (intercepts, R2 = 0.85, Q2 = −0.73 and R2 = 0.90, Q2 = −0.63) (Figure 4C,D) and that the explanatory rate (R2) of the model was greater than the predictive rate (Q2).

### 2.5. Differential Metabolite Analysis and KEGG Metabolic Pathway Enrichment

**Figure 5:**
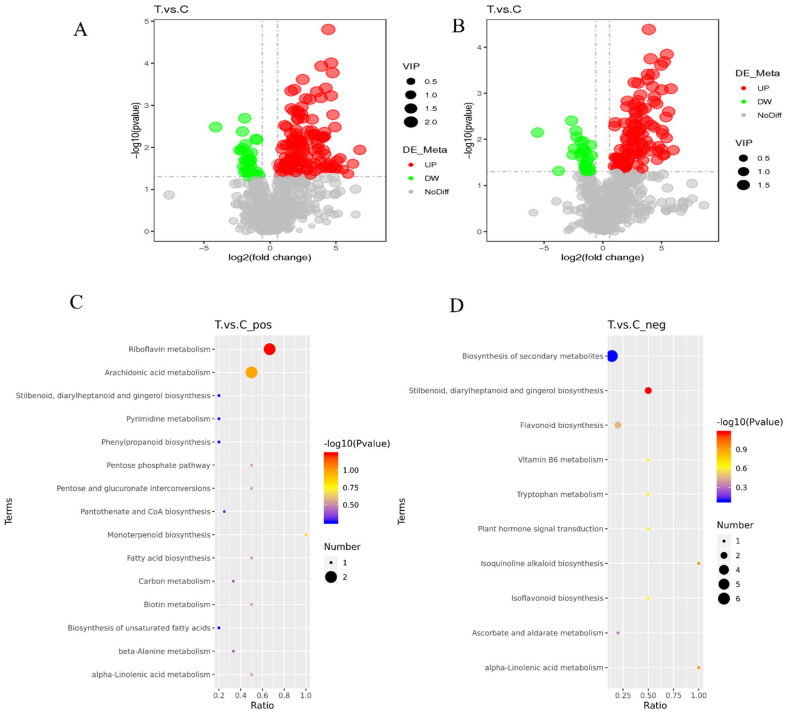
Volcano plots and KEGG metabolic pathway enrichment map of differential metabolites. (**A**,**C**) Positive ion mode, (**B**,**D**) Negative ion mode; each point represents a metabolite: horizontal coordinates indicate different multiplicities of differential metabolites (log2 values), vertical coordinates indicate *p*-values (−log10 values), grey indicates metabolites with no significant differences (NoDiff), red indicates up–regulated metabolites (UP), green indicates down–regulated metabolites (DW), and the size of the points indicates VIP values. The abscissa in the figure is x/y (the number of differential metabolites in the corresponding metabolic pathway/the total number of metabolites identified in the pathway), and the larger the value, the higher the enrichment of differential metabolites in the pathway. The color of the point represents the *p*-value of the hypergeometric test. The smaller the value, the more reliable and statistically significant the test is. The size of the dots represents the number of differential metabolites in the corresponding pathway; the larger the number, the more differential metabolites in the pathway.

To validate the key differential metabolites in the T and C samples, differential metabolites were identified by screening the VIP values of the first principal component in the PLS-DA model and the *p*-values of the *t*-test, setting thresholds of VIP > 1.0, FC > 1.5 or FC < 0.667 and *p*-value < 0.05. The identified differential metabolites were visualized using volcano plots (Figure 5A,B), and 171 and 136 differential metabolites were obtained by screening in the cationic and anionic modes, respectively. In the cationic mode, there were 137 significantly up-regulated differential metabolites and 34 significantly down-regulated differential metabolites; in the anionic mode, there were 109 significantly up-regulated differential metabolites and 27 significantly down-regulated differential metabolites.

All the differential metabolites in the different comparison groups were matched to the KEGG database to obtain the pathway information involved in the differential metabolites. Enrichment analysis was performed on the annotated results to obtain pathways with more enriched differential metabolites. In the positive mode, the differential metabolites in the T and C groups were mainly enriched in riboflavin metabolism, arachidonic acid metabolism, and monoterpenoid biosynthesis (Figure 5C). In the anion mode, the differential metabolites in the T and C groups were mainly enriched in Stilbenoid, diarylheptanoid, and gingerol biosynthesis, flavonoid biosynthesis, and the biosynthesis of secondary metabolites (Figure 5D).

### 2.6. Annotation and Analysis of LIPID MAPS

**Figure 6:**
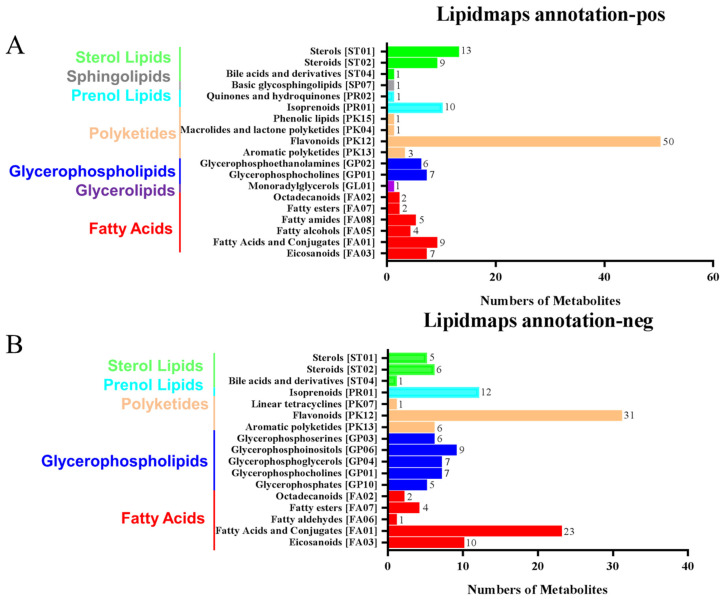

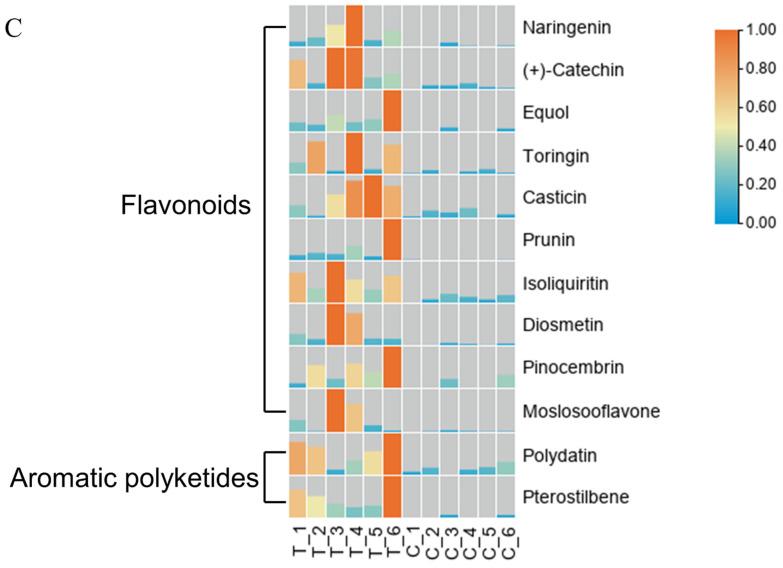
LIPID MAPS category annotation and heatmap analysis of differential metabolites of polyketides. (**A**) Positive ion mode, (**B**) Negative ion mode: the horizontal coordinate represents the number of metabolites, and the vertical coordinate represents the LIPID MAPS lipid categories annotated to; this figure shows the number of metabolites annotated to the Main_Class under the eight major lipid categories in LIPID MAPS; (**C**) Heatmap display of the different substances of polyketides.

The LIPIDMaps database http://www.lipidmaps.org/ (accessed on 9 July 2021) was used to annotate the identified differential metabolites into eight major groups, namely: (1) Fatty Acids (FA); (2) Glycerolipids (GL); (3) Glycerophospholipids (GP); (4) Sphingolipids (SP); (5) Sterol Lipids (ST); (6) Pregnenol Lipids (PR); (7) Saccharolipids (SL); (8) Polyethylene (PK). The top three metabolites for the positive ion pattern differences were 31 Polyketides (PK), 12 Prenol Lipids (PR), and 23 Fatty Acids (FA) (Figure 6A). The top three metabolites for the negative ion pattern differences were 50 Polyketides (PK), 10 Prenol Lipids (PR), and 13 Sterol Lipids (ST) (Figure 6B). We mainly focused on the content changes of Polyketides (PK) class substances because, in the positive and negative ion modes, the largest number of Polyketides (PK) species was annotated, with a total of 93 species. Among them, there are 12 different substances between the treatment group and the control group. Among them, 10 Flavonoids and 2 Aromatic polyketides were significantly elevated compared to the control group (Figure 6C).

## 3. Materials and Methods

### 3.1. Materials

Three-year-old live seedlings of *P. massoniana* were selected for inoculation in a greenhouse with essentially uniform growth. The highly virulent *B. xylophilus* strain NXY61 was isolated from wood chips of infested *P. massoniana* in Zhejiang, China and stored at the Laboratory of Forest Ecology, Environment, and Nature Conservation Institute of the Chinese Academy of Forestry, Beijing, China. The *Botrytis cinerea* strain has been maintained in our laboratory.

### 3.2. Sample Handling

After drilling holes at 45° obliquely below the branch of *P. massoniana* at 10–15 cm from the ground, 40 µL (250 individuals/µL) of nematode solution was injected into the hole with a pipette and quickly wrapped with sealing film to ensure no evaporation. Fifteen days later, samples were taken 3 cm above and below the junction between the wilted and healthy main stems with six replicates. The trunk samples were trimmed to a size of no more than 30 mm × 30 mm × 30 mm. The trimmed samples were surface sterilized with 75% ethanol for 90 s and then washed three times with sterile water. Then, the samples were peeled and cut into small, easily grindable pieces. We transferred approximately 1 g of the cut pieces into 1.5 mL tubes for storage. All the 1.5 mL test tube samples were stored in a −80 °C freezer. In total, we had 12 frozen samples for DNA extraction.

### 3.3. DNA Extraction and Amplicon Sequencing

Total genomic DNA samples were extracted using the OMEGA Soil DNA Kit (M5635-02) (OmegaBio-Tek, Norcross, GA, USA), following the manufacturer’s instructions, and stored at −20 °C prior to further analysis. The quantity and quality of extracted DNAs were measured using a NanoDrop NC2000 spectrophotometer (Thermo Fisher Scientific, Waltham, MA, USA) and agarose gel electrophoresis, respectively. PCR amplification of the bacterial 16S rRNA genes V5–V7 region was performed using the forward primer 799F (5′-AACMGGATTAGATACCCKG-3′) and the reverse primer 1193R (5′-ACGTCATCCCCACCTTCC-3′) and the ITS1 region of the fungal ITS gene using primer pairs ITSF (CTTGGTCATTTAGAGGAAGTAA) and ITS2 (GCTGCGTTCTTCATCGATGC) with the barcode. The PCR components contained 5 μL of buffer (5×), 0.25 μL of Fast pfu DNA Polymerase (5U/μL), 2 μL (2.5 mM) of dNTPs, 1 μL (10 μM) of each Forward and Reverse primer, 1 μL of DNA Template, and 14.75 μL of ddH_2_O. Thermal cycling consisted of initial denaturation at 98 °C for 5 min, followed by 25 cycles consisting of denaturation at 98 °C for 30 s, annealing at 53 °C for 30 s, and extension at 72 °C for 45 s, with a final extension of 5 min at 72 °C. PCR amplicons were purified with Vazyme VAHTSTM DNA Clean Beads (Vazyme, Nanjing, China) and quantified using the Quant-iT PicoGreen dsDNA Assay Kit (Invitrogen, Carlsbad, CA, USA). After the individual quantification step, amplicons were pooled in equal amounts, and pair-end 2 × 250 bp sequencing was performed using the Illumina NovaSeq platform with NovaSeq 6000 SP Reagent Kit (600 cycles) at Shanghai Personal Biotechnology Co., Ltd. (Shanghai, China). Raw sequences were deposited at the Sequence Read Archive (SRA) of the National Center for Biotechnology Information (NCBI) under project accession numbers PRJNA799172 and PRJNA799179.

### 3.4. Sequence Data Analysis

Microbiome bioinformatics were performed with QIIME2 2019.4 [27] with slight modifications according to the official tutorials https://docs.qiime2.org/2019.4/tutorials/ (accessed on 7 March 2021). Briefly, the raw sequence data were demultiplexed using the demux plugin followed by primers cutting with the cutadapt plugin [28]. The sequences were then quality-filtered, denoised, merged, and chimera removed using the DADA2 plugin [29]. Operational Taxonomic Units (OTUs) were aligned with mafft [30]. Taxonomy was assigned to OTUs using the classify-sklearn naive Bayes taxonomy classifier in the feature-classifier plugin [31] against the SILVA Release 132/UNITE Release 8.0 Database [32].

### 3.5. Bioinformatics and Statistical Analysis

Sequence data analyses were mainly performed using the QIIME2 and R packages (v3.2.0). OTU-level alpha diversity indices, such as the Chao1 richness estimator, Shannon diversity index, Simpson index, and Pielou’s evenness were calculated using the OTU table in QIIME2 and visualized as box plots. OTU-level ranked abundance curves were generated to compare the richness and evenness of the OTUs among samples. Beta diversity analysis was performed to investigate the structural variation of microbial communities across samples using Jaccard metrics [33], Bray–Curtis metrics [34], and UniFrac distance metrics [35,36] and visualized via principal coordinate analysis (PCoA). Principal component analysis (PCA) was also conducted based on the genus-level compositional profiles [37]. A Venn diagram was generated to visualize the shared and unique OTUs among samples or groups using the R package “VennDiagram”, based on the occurrence of OTUs across the samples/groups regardless of their relative abundance [38]. Random forest analysis was applied to discriminate the samples from different groups using QIIME2 with the default settings [39,40].

### 3.6. Metabolites Extraction

#### 3.6.1. Tissue Sample

Tissues (100 mg) were individually ground with liquid nitrogen, and the homogenate was resuspended with prechilled 80% methanol by well vortex. The samples were incubated on ice for 5 min and then were centrifuged at 15,000× *g*, 4 °C for 20 min. Some of the supernatant was diluted to a final concentration containing 53% methanol by LC-MS-grade water. The samples were subsequently transferred to a fresh Eppendorf tube and then were centrifuged at 15,000× *g*, 4 °C for 20 min. Finally, the supernatant was injected into the LC-MS/MS system analysis [41].

#### 3.6.2. UHPLC-MS/MS Analysis

UHPLC-MS/MS analyses were performed using a Vanquish UHPLC system (ThermoFisher, Bremen, Germany) coupled with an Orbitrap Q ExactiveTM HF mass spectrometer (ThermoFisher, Bremen, Germany) in Novogene Co., Ltd. (Beijing, China). The samples were injected onto a Hypesil Goldcolumn (100 × 2.1 mm, 1.9 μm) using a 17 min linear gradient at a flow rate of 0.2 mL/min. The eluents for the positive polarity mode were eluent A (0.1% FA in Water) and eluent B (Methanol). The eluents for the negative polarity mode were eluent A (5 mM ammonium acetate, pH 9.0) and eluent B (Methanol). The solvent gradient was set as follows: 2% B, 1.5 min; 2–100% B, 12.0 min; 100% B, 14.0 min; 100–2% B, 14.1 min; 2% B, 17 min. A Q ExactiveTM HF mass spectrometer was operated in positive/negative polarity mode with a spray voltage of 3.5 kV, a capillary temperature of 320 °C, a sheath gas flow rate of 35 arb, an aux gas flow rate of 10 arb, an S-lens RF level of 60, and an Aux gas heater temperature of 350 °C.

#### 3.6.3. Data Processing and Metabolite Identification

The raw data files generated by UHPLC-MS/MS were processed using Compound Discoverer 3.1 (CD3.1, ThermoFisher, Bremen, Germany) to perform peak alignment, peak picking, and quantitation for each metabolite. The main parameters were set as follows: retention time tolerance, 0.2 min; actual mass tolerance, 5 ppm; signal intensity tolerance, 30%; signal/noise ratio, 3; and minimum intensity et al. After that, peak intensities were normalized to the total spectral intensity. The normalized data were used to predict the molecular formula based on additive ions, molecular ion peaks, and fragment ions. Additionally, the peaks were then matched with the mzCloud https://www.mzcloud.org/ (accessed on 20 April 2021), mzVault, and MassList databases to obtain accurate qualitative and relative quantitative results. Statistical analyses were performed using the statistical software R version R-3.4.3: University of Auckland, New Zealand (Auckland, New Zealand), Python (Python 2.7.6 version, Amsterdam, Netherlands), and CentOS (CentOS release 6.6, Founder Gregory Kurtzer, Raleigh, North Carolina, USA), When the data were not normally distributed, normal transformations were attempted using of area normalization method.

#### 3.6.4. Data Analysis

These metabolites were annotated using the KEGG database https://www.genome.jp/kegg/pathway.html (accessed on 10 April 2021), HMDB database https://hmdb.ca/metabolites (accessed on 18 April 2021), and LIPIDMaps database http://www.lipidmaps.org/ (accessed on 10 December 2021). Principal components analysis (PCA) and partial least squares discriminant analysis (PLS-DA) were performed in metaX [42] (a flexible and comprehensive software for processing metabolomics data). We applied univariate analysis (*t*-test) to calculate the statistical significance (*p*-value). The metabolites with VIP (Variable Importance in the Projection) > 1 and *p*-value < 0.05 and fold change ≥ 2 or FC ≤ 0.5 were considered to be differential metabolites. Volcano plots were used to filter metabolites of interest, which were based on the log2 (FoldChange) and -log10 (*p*-value) of the metabolites by ggplot2 in the R language. The functions of these metabolites and metabolic pathways were studied using the KEGG database. The metabolic pathways enrichment of differential metabolites was performed; when the ratio was satisfied by x/n > y/N, the metabolic pathways were considered as enriched, and when the *p*-value of the metabolic pathways was <0.05, metabolic pathways were considered as statistically significantly enriched.

## 4. Discussion

Plants and microorganisms have always been in a love–hate relationship in the evolutionary process, and the same plant pathogen may play a positive or negative role in different plants [43,44]. During the infestation of pine trees by *B. xylophilus*, the composition of the microbial community in the pine tree will be changed. Especially, the abundance of some specific microorganisms will ultimately affect the health of the pine tree [45]. Previous studies have found that the bacteria that are toxic to nematodes are mainly species of the genera *Bacillus* [46] and *Serratia* [47], and the fungi are mainly species of the genera *Trichoderma* [17] and *Esteya* [48]. In addition, for the microbiome in pine trees infected with *B. xylophilus*, some scholars pointed out that the microbiome in the tree is closely related to plant growth, plant immune genes, and toxin biosynthesis in the microbiome [49]. However, in the pathogenesis process, which specific communities will have a key impact on the entire pathogenesis is still unknown, including whether the plant’s own metabolism will further aggravate the disease, which is also worthy of further exploration. In our study, we used LEfSe (LDA Effect Size) analysis on both groups to determine the primary characteristics that separate healthy and wilted pine communities. *Actinobacteria*, *Bacteroidia*, and *Plyosporaceae* is the key marker in wilted pines (Figure 3B,D). These Phyla might be linked to *B. xylophilus*’ pathogenicity in pines.

Many studies have focused on bacterial community changes caused by *B. xylophilus* [50,51]. Conversely, the effect of the fungal community on PWD is also quite important. First, the feeding of nematodes in the laboratory environment is mainly based on the fungus *Botrytis cinerea* as food [52]. When pathogenic fungi interact with plants, they release toxins and effector proteins that affect plant immune defenses, resulting in plant disease affecting their growth and development [53]. We discovered certain alterations in fungal communities associated with pines caused by PWD using the high-throughput Illumina MiSeq to sequence ITS data. The major fungal genera in healthy *P. massoniana* were *Didymella*, *Leucosporidium*, and *Aspergillus*. Potentially due to *B. xylophilus* infection, the abundances of *Alternaria*, *Penicillium*, and *Apiotrichum* in wilted pines were greater, and abundances of *Leucosporidium*, *Aspergillus*, and *Fusarium* were lower than compared to those in healthy pines (Figure 2D).

Zhang Wei et al., discovered that *Penicillium* was more abundant in healthy pine trees than in *B. xylophilus*-infested *P. massoniana* [45]. These variances might be attributed to changes in the nematode infection stage and the experiment sample site. Instead of a stable community composition, the community composition and dominating bacterial species in the pine tree changed dynamically with the different infection phases. This also represents the pathogenesis of PWDs’ intricacy. Through previous studies, we have noticed that when *B. xylophilus* infects pine trees for 15 days, the transcription of defense-related genes in pine trees is significantly increased [20], and the chemical defense substance α-pinene in pine trees is significantly accumulated [21]. Various phenomena have made us very interested in the key time nodes in the infestation process of *B. xylophilus*. How did the microbial community in pine trees change during the critical times of infestation? Our results showed that the fungal richness of blighted pine was higher than that of healthy pine, but the bacterial richness of blighted pine was not significantly different from that of healthy pine, suggesting that *B. xylophilus* infection may promote the reproduction of some dominant species. Examples are fungi Pleosporaceae and bacteria (Actinobacteria and Bacterodia). It is worth noting that studies have shown that *Alternaria oxytropis* (Pleosporaceae) can produce a toxic indolizidine-like secondary metabolite—swainsonine (SW). SW can inhibit animal cell α-mannose. The activity of glycosidase I and Golgi α-mannosidase II can degenerate cell vacuoles, lose normal function, and even die in severe poisoning [54,55,56]. In terms of the emergence of dominant populations due to changes in the microbial community, and the potential to metabolize some compounds that are harmful to the host, how much does this compound act in the pathogenic process of *B. xylophilus*? We still need to further study the host metabolism of this key node.

To date, metabolites of many key species have been successfully analyzed using metabolomics [57,58,59]. Previous studies on pine metabolites have focused on metabolic differences in different environments such as altitude [60], drought stress [61], and vector insect induction [62]. However, there is no detailed study on the differences in host metabolic characteristics during the pathogenesis of *B. xylophilus*. In this study, we used broad-targeted LC-MS/MS-based metabolomics to understand differences in stem metabolites during *B. xylophilus* pathogenesis. We identified a total of 307 differential metabolites, of which, 246 were significantly up-regulated and 61 were significantly down-regulated (Figure 5). Through KEGG enrichment analysis and differential metabolite annotation analysis, it was found that polyethylene metabolites accumulated significantly in withered pine trees. After relative content analysis, there were 12 core metabolites, including 10 flavonoids metabolites, and 2 kinds of aromatic polyketide metabolites. Flavonoids are closely related to plant immunity, e.g., soybean [63], tomato [64], and apple [65]. Similar to this, when pine trees are infected by the pine woodworm, flavonoid compounds are crucial to the pine plants’ defense mechanisms. Researchers discovered that DEGs greatly enhanced the biological processes associated with phenylpropane biosynthesis, flavonoid biosynthesis, redox, and plant-type hypersensitivity by the transcriptome analysis of pine trees infected with pine wood nematodes [66]. Additionally, Qiaoli Chen et al., discovered that the majority of the body’s stress resistance genes were adversely downregulated when pine trees were infected with pine wood nematode [67]. However, the shift in the nematode population is directly correlated with the expression of the chalcone synthase genes, which are strongly associated with the manufacture of flavonoids, flavonoids and flavonols, and phenylpropane. This demonstrates that during the early stages of pine wood nematode infection, the host pine will begin to metabolize certain defensive chemicals in the tree. Further research into these metabolites will yield crucial information for the early identification of PWN.

Although many studies have paid more attention to the inhibitory effect of plant metabolites on pathogenic bacteria, for *B. xylophilus*, pathogenic factors such as disease season, vector insects, transmission conditions, and host metabolism need to be considered. It is worth emphasizing that when specific metabolites accumulate abnormally, metabolic problems within the plant itself can exacerbate disease and accelerate plant death. Including those mentioned above, the dominant family, Pleosporaceae, metabolizes some substances that are harmful to the host, but no SW was found from our metabolome data. Perhaps it was because the dominant bacteria had just reproduced on the 15th day, and no further toxic substances had been secreted to exacerbate the disease. Therefore, our follow-up plan focuses on isolating the dominant strains after nematode invasion and focusing on the effects of their metabolites on the pathogenicity of *B. xylophilus* through purification and culture. Finally, this study provides new evidence for a potential pathogenic cause of pine wood nematode.

## Figures and Tables

**Table 1:** Richness of bacterial communities of healthy and *B. xylophilus*–infected *P. massoniana*. Nematode-infected *P. massoniana* is defined as wilted pines.

Samples		Species Richness Indices
Location	Host Status	OTUs Observed
Endophytic bacteria (EB)	Wilted pines	160.40 ± 35.78
	Healthy pines	199.00 ± 104.60
Endophytic fungi (EF)	Wilted pines	49.80 ± 25.08
	Healthy pines	72.20 ± 42.59

Mean ± SD.

## Data Availability

The data used in this study are available from the corresponding author on submission of a reasonable request.

## References

[1] Vicente C., Espada M., Vieira P., Mota M. (2012). Pine Wilt Disease: A threat to European forestry. Eur. J. Plant Pathol..

[2] Kojima K., Kamijyo A., Masumori M., Sasaki S. (1994). Cellulase activities of pine-wood nematode isolates with different virulences. J. Jpn. For. Soc..

[3] Odani K., Sasaki S., Nishiyama Y., Yamamoto N. (2008). Early Symptom Development of the Pine Wilt Disease by Hydrolytic Enzymes Produced by the Pine Wood Nematodes. Drug Saf..

[4] Myers R.F. (1986). Cambium destruction in conifers caused by pinewood nematodes. J. Nematol..

[5] Kuroda K. (2002). The mechanism oftra—Cheid cavitation in trees infected with wilt diseases. Proc. IUFRO Work. Party.

[6] Kawazu K., Zhang H., Kanzaki H. (1996). Accumulation of benzoic acid in suspension cultured cells of *Pinus thunbergii* Parl. in response to phenylacetic acid administration. Biosci. Biotechnol. Biochem..

[7] Oku H. (1990). Phytotoxins in pine (*Pinus* spp.) wilt disease Bursaphelenchus xyophilus. Nippon. Nogeik Kaishi.

[8] Oku H. (1988). Role ofphytotoxins in pine wilt disease. J. Nematol..

[9] Wang J., Han S., Li Y., Deng X., Zhang X. (2014). Cloning of TLP-1 Gene and Prediction of TLP-1 Protein Structure of *Bursaphelenchus xylophilus*. J. Sichuan Agric. Univ..

[10] Dang Q.L., Son S.W., Cheon H.-M., Choi G.J., Choi Y.H., Jang K.S., Lim C.H., Kim J.-C. (2011). Pyochelin isolated from Burkholderia arboris KRICT1 carried by pine wood nematodes exhibits phytotoxicity in pine callus. Nematology.

[11] Oku H., Shiraishi T., Ouchi S., Kurozumi S., Ohta H. (1980). Pine Wilt Toxin, the Metabolite of a Bacterium Associated with a Nematode. Naturwissenschaften.

[12] Nascimento F.X., Espada M., Barbosa P., Rossi M.J., Vicente C.S.L., Mota M. (2016). Non-specific transient mutualism between the plant parasitic nematode, *Bursaphelenchus xylophilus*, and the opportunistic bacterium *Serratia* quinivorans BXF1, a plant-growth promoting pine endophyte with antagonistic effects. Environ. Microbiol..

[13] Zhao B., Liu Y., Lin F. (2006). Mutual influences in growth and reproduction between pine wood nematode *Bursaphelenchus xylophilus* and bacteria it carries. Front. For. China.

[14] Pires D., Vicente C.S.L., Inacio M.L., Mota M. (2022). The Potential of *Esteya* spp. for the Biocontrol of the Pinewood Nematode, *Bursaphelenchus xylophilus*. Microorganisms.

[15] Marques-Pereira C., Proenca D.N., Morais P.V. (2022). The Role of Serratomolide-like Amino Lipids Produced by Bacteria of Genus *Serratia* in Nematicidal Activity. Pathogens.

[16] Maehara N. (2008). Reduction of *Bursaphelenchus xylophilus* (Nematoda: Parasitaphelenchidae) population by inoculating *Trichoderma* spp. into pine wilt-killed trees. Biol. Control.

[17] Ni Z., Zhou P., Xu M., Xu L.-A. (2018). Development and characterization of chloroplast microsatellite markers for *Pinus massoniana* and their application in *Pinus* (Pinaceae) species. J. Genet..

[18] Liu Q., Wei Y., Xu L., Hao Y., Chen X., Zhou Z. (2017). Transcriptomic Profiling Reveals Differentially Expressed Genes Associated with Pine Wood Nematode Resistance in Masson Pine (*Pinus massoniana* Lamb.). Sci. Rep..

[19] Meng F., Li Y., Liu Z., Wang X., Feng Y., Zhang W., Zhang X. (2020). Potential Molecular Mimicry Proteins Responsive to α-pinene in *Bursaphelenchus xylophilus*. Int. J. Mol. Sci..

[20] Gershenzon J. (1994). Metabolic costs of terpenoid accumulation in higher plants. J. Chem. Ecol..

[21] Turlings T.C., Loughrin J.H., McCall P.J., Rose U.S., Lewis W.J., Tumlinson J.H. (1995). How caterpillar-damaged plants protect themselves by attracting parasitic wasps. Proc. Natl. Acad. Sci. USA.

[22] Chao A. (1984). Nonparametric-Estimation of the Number of Classes in a Population. Scand. J. Stat..

[23] Shannon C.E. (1948). A mathematical theory of communication. Bell Syst. Tech. J..

[24] Simpson E.H. (1949). Measurement of Diversity. Nature.

[25] Pielou E.C. (1966). The measurement of diversity in different types of biological collections. J. Theor. Biol..

[26] Bolyen E., Rideout J.R., Dillon M.R., Bokulich N., Abnet C.C., Al-Ghalith G.A., Alexander H., Alm E.J., Arumugam M., Asnicar F. (2019). Reproducible, interactive, scalable and extensible microbiome data science using QIIME 2. Nat. Biotechnol..

[27] Martin M. (2011). Cutadapt removes adapter sequences from high-throughput sequencing reads. EMBnet.

[28] Callahan B.J., McMurdie P.J., Rosen M.J., Han A.W., Johnson A.J.A., Holmes S.P. (2016). DADA2: High-resolution sample inference from Illumina amplicon data. Nat. Methods.

[29] Katoh K., Misawa K., Kuma K., Miyata T. (2002). MAFFT: A novel method for rapid multiple sequence alignment based on fast Fourier transform. Nucleic Acids Res..

[30] Bokulich N.A., Kaehler B.D., Rideout J.R., Dillon M., Bolyen E., Knight R., Huttley G.A., Caporaso J.G. (2018). Optimizing taxonomic classification of marker-gene amplicon sequences with QIIME 2’s q2-feature-classifier plugin. Microbiome.

[31] Koljalg U., Nilsson R.H., Abarenkov K., Tedersoo L., Taylor A.F.S., Bahram M., Bates S.T., Bruns T.D., Bengtsson-Palme J., Callaghan T.M. (2013). Towards a unified paradigm for sequence-based identification of fungi. Mol. Ecol..

[32] Jaccard P. (1908). Nouvelles recherches sur la distribution florale. Bull. Soc. Vaud. Des Sci. Nat..

[33] Bray J.R., Curtis J.T. (1957). An ordination of the upland forest communities of southern wisconsin. Ecol. Monogr..

[34] Lozupone C., Knight R. (2005). UniFrac: A new phylogenetic method for comparing microbial communities. Appl. Environ. Microbiol..

[35] Lozupone C.A., Hamady M., Kelley S.T., Knight R. (2007). Quantitative and qualitative beta diversity measures lead to different insights into factors that structure microbial communities. Appl. Environ. Microbiol..

[36] Ramette A. (2007). Multivariate analyses in microbial ecology. Fems Microbiol. Ecol..

[37] Zaura E., Keijser B.J.F., Huse S.M., Crielaard W. (2009). Defining the healthy “core microbiome” of oral microbial communities. BMC Microbiol..

[38] Breiman L. (2001). Random forests. Mach. Learn..

[39] Liaw A., Wiener M.C. (2002). Classification and Regression by randomForest. R News.

[40] Want E.J., Masson P., Michopoulos F., Wilson I.D., Theodoridis G., Plumb R.S., Shockcor J., Loftus N., Holmes E., Nicholson J.K. (2013). Global metabolic profiling of animal and human tissues via UPLC-MS. Nat. Protoc..

[41] Wen B., Mei Z., Zeng C., Liu S. (2017). Metax: A flexible and comprehensive software for processing metabolomics data. BMC Bioinform..

[42] Han G., Mannaa M., Kim N., Jeon H.W., Jung H., Lee H.H., Kim J., Park J., Park A.R., Kim J.C. (2021). Response of Pine Rhizosphere Microbiota to Foliar Treatment with Resistance-Inducing Bacteria against Pine Wilt Disease. Microorganisms.

[43] Proença D.N., Francisco R., Kublik S., Schöler A., Vestergaard G., Schloter M., Morais P.V. (2017). The Microbiome of Endophytic, Wood Colonizing Bacteria from Pine Trees as Affected by Pine Wilt Disease. Sci. Rep..

[44] Zhang W., Wang X., Li Y., Liu Z., Li D., Wen X., Feng Y., Zhang X. (2020). Pinewood Nematode Alters the Endophytic and Rhizospheric Microbial Communities of *Pinus massoniana*. Microb. Ecol..

[45] Li L., Tan J., Chen F. (2018). *Bacillus pumilus* strain LYMC-3 shows nematicidal activity against *Bursaphelenchus xylophilus* via the production of a guanidine compound. Biocontrol Sci. Technol..

[46] Proença D.N., Santo C.E., Grass G., Morais P.V. (2012). Draft Genome Sequence of *Serratia* sp. Strain M24T3, Isolated from Pinewood Disease Nematode *Bursaphelenchus xylophilus*. J. Bacteriol..

[47] Wang Z., Zhang Y., Wang C., Wang Y., Sung C. (2017). Esteya vermicola controls the pinewood nematode, *Bursaphelenchus xylophilus*, in pine seedlings. Econ. Res.-Ekon. Istraživanja.

[48] Alves M., Pereira A., Vicente C., Matos P., Henriques J., Lopes H., Nascimento F., Mota M., Correia A., Henriques I. (2018). The role of bacteria in pine wilt disease: Insights from microbiome analysis. FEMS Microbiol. Ecol..

[49] Ma Y., Qu Z.L., Liu B., Tan J.J., Asiegbu F.O., Sun H. (2020). Bacterial Community Structure of *Pinus thunbergii* Naturally Infected by the Nematode *Bursaphelenchus xylophilus*. Microorganisms.

[50] Xue Q., Xiang Y., Wu X.-Q., Li M.-J. (2019). Bacterial Communities and Virulence Associated with Pine Wood Nematode *Bursaphelenchus xylophilus* from Different *Pinus* spp.. Int. J. Mol. Sci..

[51] Zhang W., Zhao L., Zhou J., Yu H., Zhang C., Lv Y., Lin Z., Hu S., Zou Z., Sun J. (2019). Enhancement of oxidative stress contributes to increased pathogenicity of the invasive pine wood nematode. Philos. Trans. R. Soc. B Biol. Sci..

[52] Han L.-B., Li Y.-B., Wang F.-X., Wang W.-Y., Liu J., Wu J.-H., Zhong N.-Q., Wu S.-J., Jiao G.-L., Wang H.-Y. (2019). The Cotton Apoplastic Protein CRR1 Stabilizes Chitinase 28 to Facilitate Defense against the Fungal Pathogen Verticillium dahliae. Plant Cell.

[53] James L.F., Hartley W.J., Van Kampen K.R. (1981). Syndromes of astragalus poisoning in livestock. J. Am. Vet. Med. Assoc..

[54] Braun K., Romero J., Liddell C., Creamer R. (2003). Production of swainsonine by fungal endophytes of locoweed. Mycol. Res..

[55] Grum D.S., Cook D., Baucom D., Mott I.W., Gardner D.R., Creamer R., Allen J.G. (2013). Production of the Alkaloid Swainsonine by a Fungal Endophyte in the HostSwainsona canescens. J. Nat. Prod..

[56] Ghallab D.S., Shawky E., Ibrahim R.S., Mohyeldin M.M. (2022). Comprehensive metabolomics unveil the discriminatory metabolites of some Mediterranean Sea marine algae in relation to their cytotoxic activities. Sci. Rep..

[57] Salvatore M.M., Di Lelio I., DellaGreca M., Nicoletti R., Salvatore F., Russo E., Volpe G., Becchimanzi A., Mahamedi A.E., Berraf-Tebbal A. (2022). Secondary Metabolites, including a New 5,6-Dihydropyran-2-One, Produced by the Fungus Diplodia corticola. Aphicidal Activity of the Main Metabolite, Sphaeropsidin A. Molecules.

[58] Tohge T., Borghi M., Fernie A.R. (2018). The natural variance of the Arabidopsis floral secondary metabolites. Sci. Data.

[59] Mullin M., Klutsch J.G., Cale J.A., Hussain A., Zhao S., Whitehouse C., Erbilgin N. (2021). Primary and Secondary Metabolite Profiles of Lodgepole Pine Trees Change with Elevation, but Not with Latitude. J. Chem. Ecol..

[60] Castander-Olarieta A., Montalbán I.A., De Medeiros Oliveira E., Dell’Aversana E., D’Amelia L., Carillo P., Steiner N., Fraga H.P.D.F., Guerra M.P., Goicoa T. (2019). Effect of Thermal Stress on Tissue Ultrastructure and Metabolite Profiles During Initiation of Radiata Pine Somatic Embryogenesis. Front. Plant Sci..

[61] Cale J.A., Klutsch J.G., Dykstra C.B., Peters B., Erbilgin N. (2019). Pathophysiological responses of pine defensive metabolites largely lack differences between pine species but vary with eliciting ophiostomatoid fungal species. Tree Physiol..

[62] Bentivenha J.P.F., Canassa V.F., Baldin E.L.L., Borguini M.G., Lima G.P.P., Lourencao A.L. (2018). Role of the Rutin and Genistein Flavonoids in Soybean Resistance to Piezodorus guildinii (Hemiptera: Pentatomidae). Arthropod-Plant Interact..

[63] Yao Q., Peng Z., Tong H., Yang F., Xing G., Wang L., Zheng J., Zhang Y., Su Q. (2019). Tomato Plant Flavonoids Increase Whitefly Resistance and Reduce Spread of Tomato yellow leaf curl virus. J. Econ. Entomol..

[64] Lu Y., Chen Q., Bu Y., Luo R., Hao S., Zhang J., Tian J., Yao Y. (2017). Flavonoid Accumulation Plays an Important Role in the Rust Resistance of Malus Plant Leaves. Front. Plant Sci..

[65] Lee I.H., Han H., Koh Y.H., Kim I.S., Lee S.-W., Shim D. (2019). Comparative Transcriptome Analysis of *Pinus densiflora* Following Inoculation with Pathogenic (*Bursaphelenchus xylophilus*) or Non-pathogenic Nematodes (*B. thailandae*). Sci. Rep..

[66] Chen Q., Zhang R., Li D., Wang F. (2021). Transcriptomic and Coexpression Network Analyses Revealed Pine Chalcone Synthase Genes Associated with Pine Wood Nematode Infection. Int. J. Mol. Sci..

[67] Abelleira A., Picoaga A., Mansilla J.P., Aguin O. (2011). Detection of *Bursaphelenchus xylophilus*, Causal Agent of Pine Wilt Disease on *Pinus pinaster* in Northwestern Spain. Plant Dis..

